# Identification and validation of an individualized autophagy-clinical prognostic index in gastric cancer patients

**DOI:** 10.1186/s12935-020-01267-y

**Published:** 2020-05-20

**Authors:** Jieping Qiu, Mengyu Sun, Yaoqun Wang, Bo Chen

**Affiliations:** 1 Department of Clinical Medicine, The First Clinical College, Anhui Medical University, Hefei, China; 2 Department of Gastrointestinal Surgery Center, The First Affiliated Hospital of Anhui Medical University, NO. 218 Jixi Road, Hefei, Anhui 230000 China

**Keywords:** Gastric cancer, Autophagy, Prognosis, Bioinformatics

## Abstract

**Background:**

The purpose of this study is to perform bioinformatics analysis of autophagy-related genes in gastric cancer, and to construct a multi-gene joint signature for predicting the prognosis of gastric cancer.

**Methods:**

GO and KEGG analysis were applied for differentially expressed autophagy-related genes in gastric cancer, and PPI network was constructed in Cytoscape software. In order to optimize the prognosis evaluation system of gastric cancer, we established a prognosis model integrating autophagy-related genes. We used single factor Cox proportional risk regression analysis to screen genes related to prognosis from 204 autophagy-related genes in The Atlas Cancer Genome (TCGA) gastric cancer cohort. Then, the generated genes were applied to the Least Absolute Shrinkage and Selection Operator (LASSO). Finally, the selected genes were further included in the multivariate Cox proportional hazard regression analysis to establish the prognosis model. According to the median risk score, patients were divided into high-risk group and low-risk group, and survival analysis was conducted to evaluate the prognostic value of risk score. Finally, by combining clinic-pathological features and prognostic gene signatures, a nomogram was established to predict individual survival probability.

**Results:**

GO analysis showed that the 28 differently expressed autophagy-related genes was enriched in cell growth, neuron death, and regulation of cell growth. KEGG analysis showed that the 28 differently expressed autophagy-related genes were related to platinum drug resistance, apoptosis and p53 signaling pathway. The risk score was constructed based on 4 genes (GRID2, ATG4D,GABARAPL2, CXCR4), and gastric cancer patients were significantly divided into high-risk and low-risk groups according to overall survival. In multivariate Cox regression analysis, risk score was still an independent prognostic factor (HR = 1.922, 95% CI = 1.573–2.349, P < 0.001). Cumulative curve showed that the survival time of patients with low-risk score was significantly longer than that of patients with high-risk score (P < 0.001). The external data GSE62254 proved that nomograph had a great ability to evaluate the prognosis of individual gastric cancer patients.

**Conclusions:**

This study provides a potential prognostic marker for predicting the prognosis of GC patients and the molecular biology of GC autophagy.

## Background

Gastric cancer (GC) is a common disease that threatens human health. It is composed of adenocarcinoma, squamous cell carcinoma, adenosquamous carcinoma, carcinoid, etc. of which gastric adenocarcinoma accounts for the vast majority. In 2018, more than 1,000,000 people were diagnosed with gastric cancer, which caused about 783,000 deaths (equivalent to 1 in every 12 deaths worldwide), making it the fifth most frequently diagnosed cancer and the third major cause of cancer death [1]. The prognosis of gastric cancer is related to pathological stage, location, tissue type, biological behavior and treatment. [2]. Up to now, histologic diagnosis and TNM staging are still the main methods to evaluate the prognosis of gastric cancer [3]. However, the existing evaluation indicators can not cover all the disease information of patients, and can not be used to accurately predict the prognosis of GC patients. Therefore, it is necessary to explore effective prognostic biomarkers to help optimize the prognosis evaluation system of gastric cancer. In the past few decades, people have learned more and more about the characteristics of tumors. One of the breakthroughs is the participation of autophagy process in the development of cancer [4–6].

Autophagy refers to the physiological and pathological process that relies on the lysosomes of cells to degrade or remove excess or damaged organelles, fold wrong proteins and invading microorganisms [7]. Autophagy widely exists in eukaryotic cells, which is one of the necessary ways of organelle renewal and metabolism dynamic balance in the organic body. It maintains the homeostasis of the intracellular environment at the basic level under the normal physiological state. When the body encounters hunger, growth factor deficiency, oxidative stress, injury and other events, the range of autophagy may raise dramatically, that is, megaautophagy, to provide nutrition and clean harmful substances [8]. Some signal transduction pathways are involved in autophagy regulation, such as mTOR signaling pathway, PI3K/Akt/(PKB) pathway, ras-raf-1-mek-erk1/2 signaling pathway [9].

Autophagy plays an intricate and contradictory role in all stages of tumor development. On the one hand, basic autophagy in normal cells can avoid the accumulation of some DNA mutation molecules and organelles, and prevent cell transformation [10]. On the other hand, the long-term upregulation of basal autophagy in tumor cells is conducive to the cancer cells being in a state of aging or dormancy, such as tumor stem cells, which will enhance the tolerance of tumor to chemotherapy or radiotherapy, and increase the recurrence rate of tumor. [11]. More and more studies have shown the significance of autophagy in gastric cancer. Beclin-1 is a key regulator of autophagy. Ahn et al. Studied the protein expression level of beclin-1 in 60 cases of gastric cancer, found that 83% of gastric cancer had beclin-1 expression, but almost no beclin-1 expression in normal gastric mucosa cells [12]. Kang et al. Also reached the same conclusion [13]. ATG5 is a key autophagy related protein. Ge et al. found that the high expression of ATG5 was significantly related to poor overall survival (OS) and disease-free survival (DFS) in gastric cancer patients [14]. In addition, the autophagy marker protein LC3 is also associated with low prognosis of gastric cancer [15]. These findings confirm the relationship between autophagy and gastric cancer, and indicate the great potential of autophagy related genes as a prognostic marker of gastric cancer.

In order to explore autophagy-related genes in gastric cancer, we analyzed the differentially expressed autophagy-related genes in gastric cancer by GO and KEGG analysis, and constructed PPI network map. In order to optimize the prognosis evaluation system of gastric cancer, we established a prognosis model integrating autophagy related genes of gastric cancer. We used single factor Cox proportional risk regression analysis to screen genes related to prognosis from 204 autophagy related genes in The Atlas Cancer Genome (TCGA) gastric cancer dataset. Then, the generated genes were applied to the Least Absolute Shrinkage and Selection Operator (LASSO). Finally, the selected genes were further included in the multivariate Cox proportional risk regression analysis to establish the prognosis model. According to the median risk score, patients were divided into high-risk group and low-risk group, and survival analysis was conducted to evaluate the prognostic value of risk score. Finally, by combining clinical-pathological features and prognostic gene signatures, a nomogram was established to predict individual survival probability.

## Methods

### Data acquisition

Search for the word “autophagy” on the Human Autophagy-dedicated Database(HADb) (http://www.autophagy.lu) to retrieve autophagy related genes. The original RNA seq data set and clinical features of the TCGA gastric dataset can be downloaded from the TCGA website (https://portal.gdc.cancer.gov/). Use R (version 3.6.0) software to standardize and process data. GSE62254 data set was obtained from Gene Expression Omnibus (GEO, https://www.ncbi.nlm.nih.gov/geo/) for validation.

### Identification and enrichment analysis of DE-ATGs

The R package “clusterprofiler” was used to carry out Gene Ontology (GO) enrichment analysis including biological process(BP), cell components(CC) and molecular functions(MF) for the differentially expressed autophagy-related genes(DE-ATGs). The same tool is also used for the enrichment analysis of Kyoto Encyclopedia of Genes and Genomes (KEGG) enrichment analysis. Then, we use string database (https://string-db.org/) to construct protein–protein interaction (PPI) network of autophagy related genes, and import the data into the software of Cytoscape to visualize the interaction of PPI network.

### Identification and verification of prognostic gene signatures

Single variable Cox proportional risk regression analysis was performed to screen autophagy-related genes (ATGs) significantly associated with overall survival (OS) in the TCGA gastric cancer dataset. The OS related genes identified were included in the LASSO regression analysis by using the R package “glmnet” to screen the genes. Then, the multivariable Cox proportional risk regression analysis was carried out to establish the prognosis model of gastric cancer ATGs. We used the following formula to calculate the risk score of each patient: risk score = ∑ X J * coef J, where coef J is coefficient, and X J is relative expression level of each ATG standardized by Z-score. The median risk score was determined as the critical value to divide the STAD dataset into high risk and low risk. In order to determine the role of risk score in predicting the clinical prognosis of GC patients, Kaplan–Meier Plotter was drawn to analyze the different survival time between high-risk group and low-risk group.

In order to study whether autophagy related risk index can be used as an independent predictor of OS in TCGA dataset of GC patients, single variable and multivariate Cox regression analysis were conducted. Risk score, age, gender, tumor subtype, pathological stage and histological grade were used as covariates.

### The construction of nomogram

Age, gender, stage, grade, T, N, M, and risk score were used to construct the nomogram together using the “rms” and “survival” packages in R. Then, calibration curves were drawn to assess the consistency between actual and predicted survival.

## Results

### Identification and enrichment analysis of DE-ATGs

**Fig. 1:**
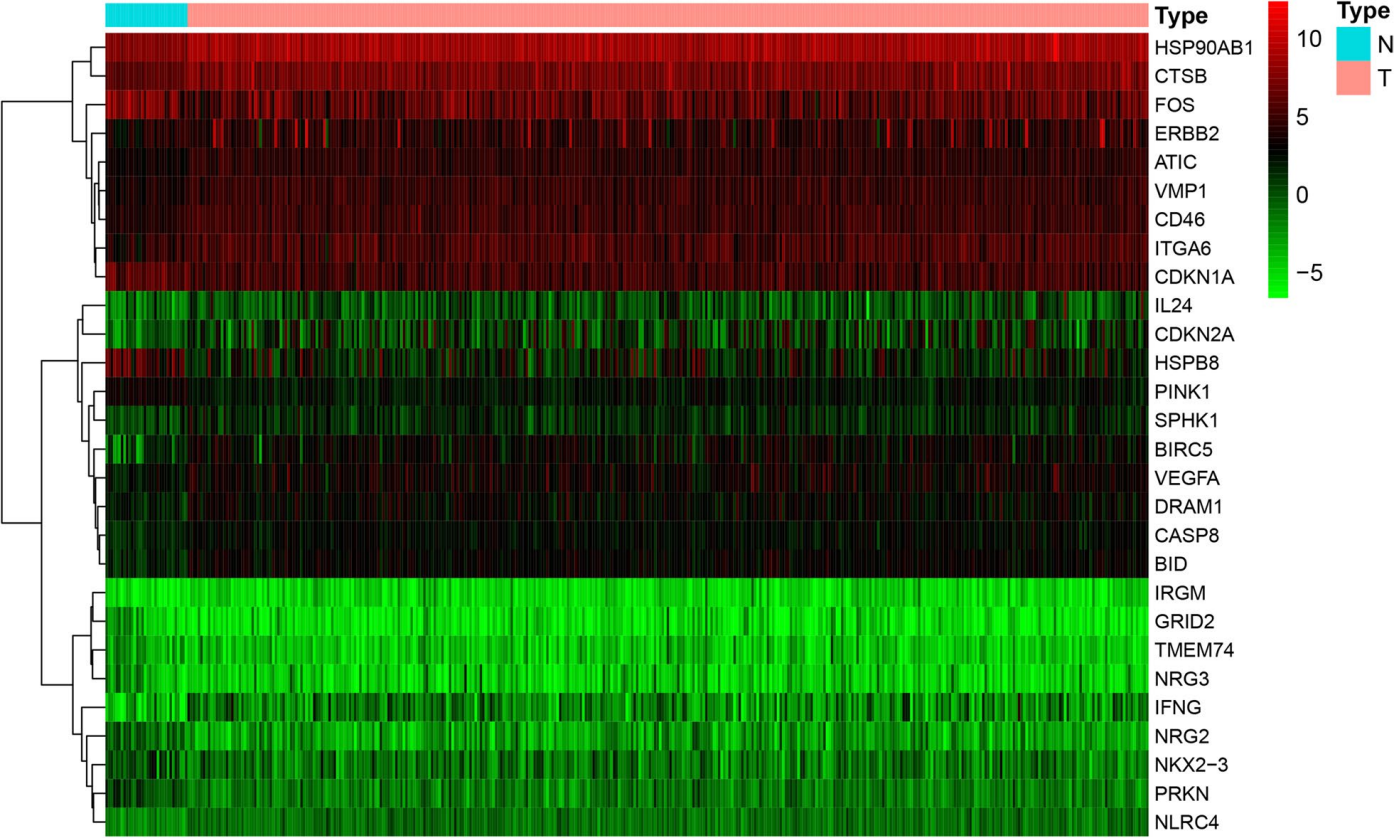
Heatmap of the expression levels of 28 DE-ATGs in TCGA-STAD. *N* normal; *T* Tumor; *Red* upregulation; *Green* downregulation. The value of expression intensity are based on the gene expression level analysis by R software.

**Fig. 2:**
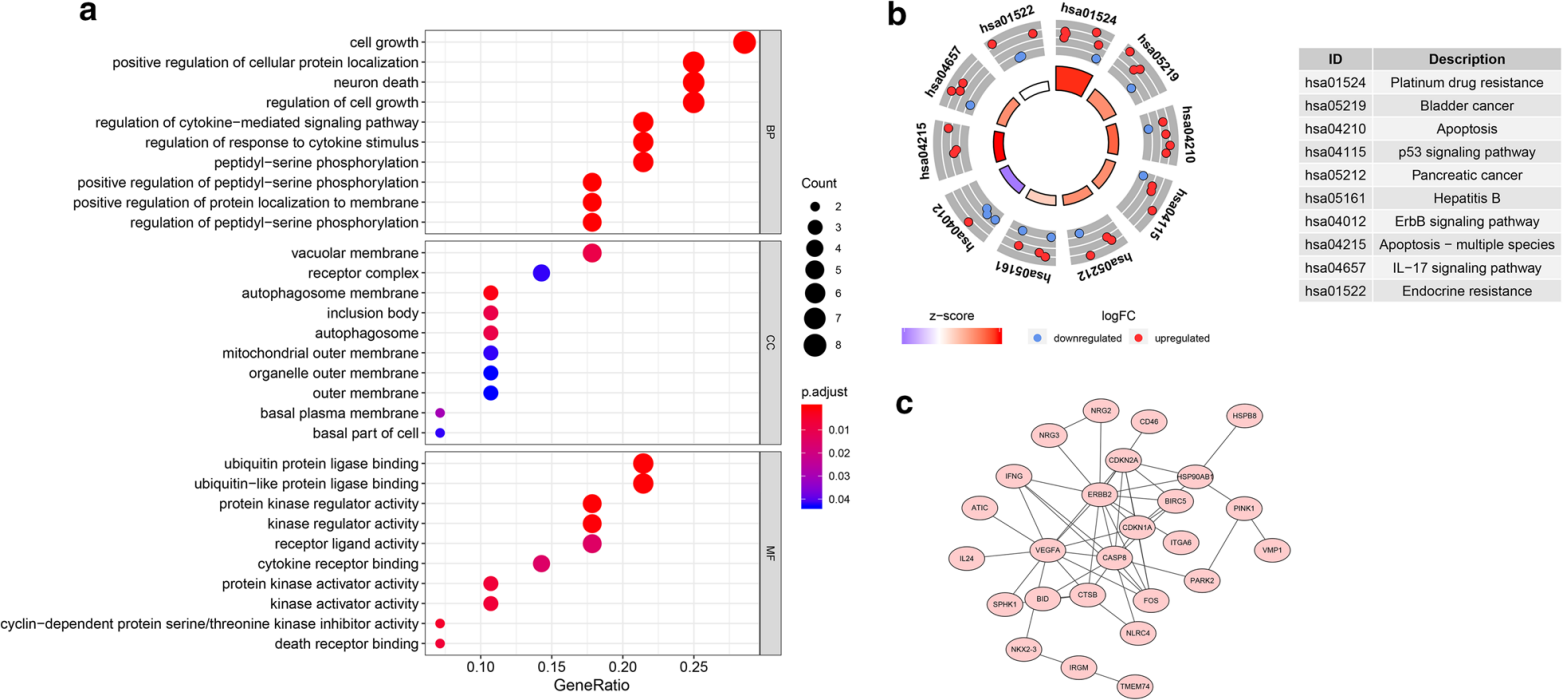
GO, KEGG enrichment analysis and PPI network. **a** GO analysis of 28 differentially expressed autophagy-related genes. “BP” stands for “biological process”, “CC” stands for “cellular component” and “MF” stands for “molecular function”. **b** KEGG analysis of 28 differentially expressed autophagy-related genes. **c** PPI network diagram of 28 differentially expressed autophagy-related genes

The TCGA-STAD cohort consisted of 407 cases, including 375 patients and 32 normal cases. Clinico-pathologic features of the patients in TCGA-STAD cohort were showed in Additional file 1. We searched for ATGs in the HADB database. A total of 232 ATGs were selected (Additional file 2), 204 of which were expressed in TCGA gastric dataset. In order to inquire about the potential signal pathways related to 204 autophagy related genes in gastric cancer, we screened them and analyzed them with GO and KEGG. 204 autophagy related genes were screened by R-packet “limma”, and the screening criteria were | lgfc | > 2, and adj. *P* < 0.05. Results showed 28 ATGs were differentially expressed in TCGA-STAD dataset (Fig. 1). GO analysis shows that these ATGs can be enriched in several basic biological processes(BP), including cell growth, positive regulation of cell protein localization, neuron death, regulation of cell growth (Fig. 2a). KEGG analysis showed that the 28 ATGs were mainly related to autophagy, platinum drug resistance, apoptosis, and p53 signaling pathway (Fig. 2b). These genes are linked to form a protein protein interaction (PPI) network, as shown in Fig. 2c (https://string-db.org/).

### Construction of prognostic markers in TCGA gastric cancer dataset

**Fig. 3:**
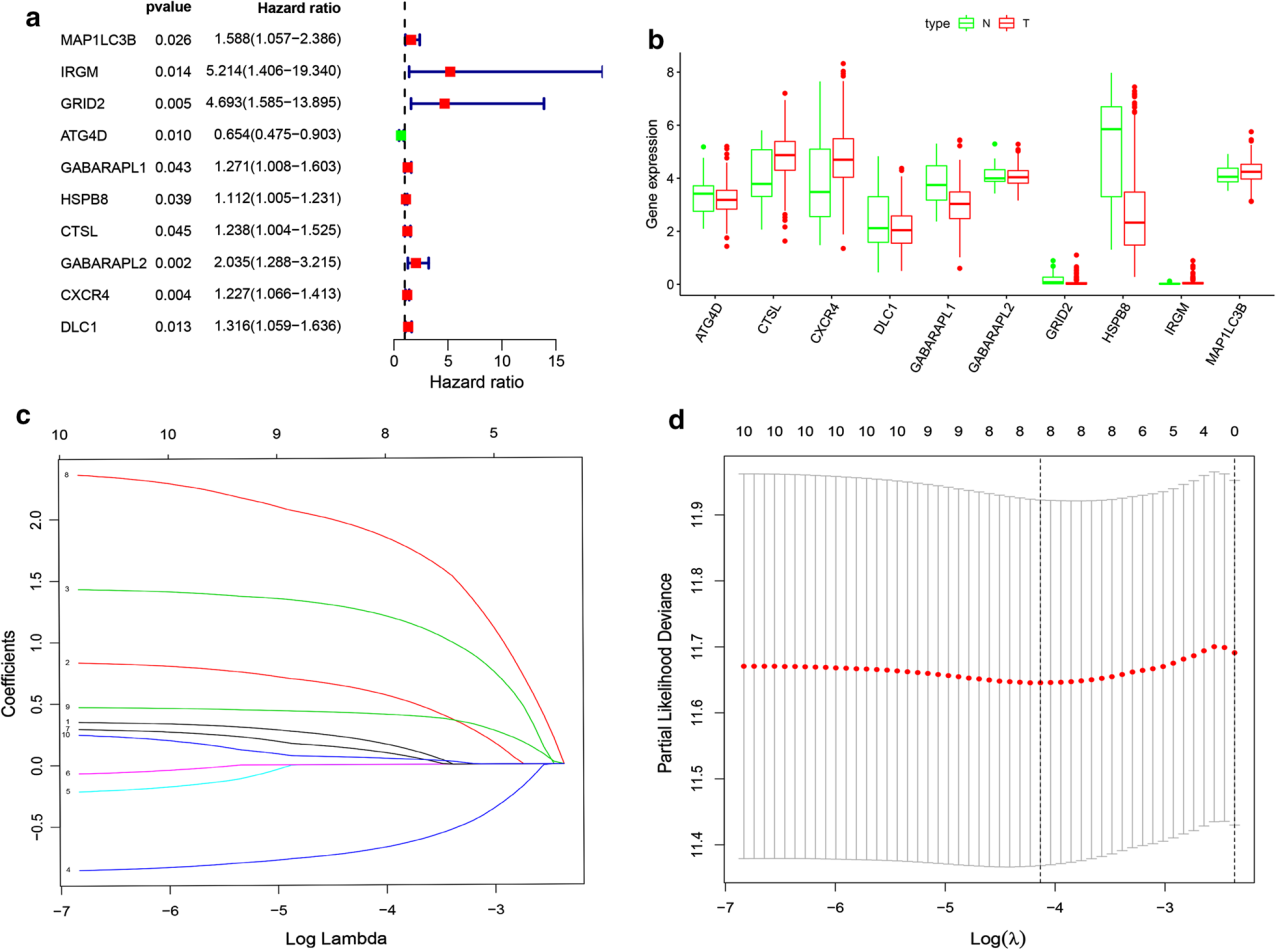
Regression analysis to select autophagy genes related to prognosis of gastric cancer. **a** Forest map of autophagy genes related to STAD survival, analyzed by univariate Cox regression. **b** Boxplot of autophagy genes associated with STAD survival, analyzed by LASSO regression.”N” stands for “normal” and “T” stands for “Tumor”. **c** LASSO coefficient spectrum of 10 genes in STAD. Generate a coefficient distribution map for a logarithmic (λ) sequence. **d** Selecting the best parameters for STAD in the LASSO model (λ)

204 ATGs were analyzed by single variable Cox regression. There are 10 genes associated with TCGA-STAD (Fig. 3a). These important genes entered the LASSO regression analysis, although including 8 genes, the model obtained the best performance (Fig. 3b–d). The 8 genes were further analyzed by multivariate Cox regression analysis, and finally 4 genes (GRID2, ATG4D, GABARAPL2 and CXCR4) related to the prognosis of STAD were obtained. The coefficients of each gene are shown in Table 1.

**Table 1:** Genes included in prognostic gene signature

Gene symbol	Full name	Coefficient	HR	P value
GRID2	Glutamate ionotropic receptor delta type subunit 2	1.865679589	6.460324759	0.001034234
ATG4D	Autophagy related 4D cysteine peptidase	− 0.378353964	0.684987997	0.021107318
GABARAPL2	GABA type A receptor associated protein like 2	0.6654797	1.945423518	0.006096631
CXCR4	C–X–C Motif chemokine receptor 4	0.158333802	1.171557197	0.028902714

**Fig. 4:**
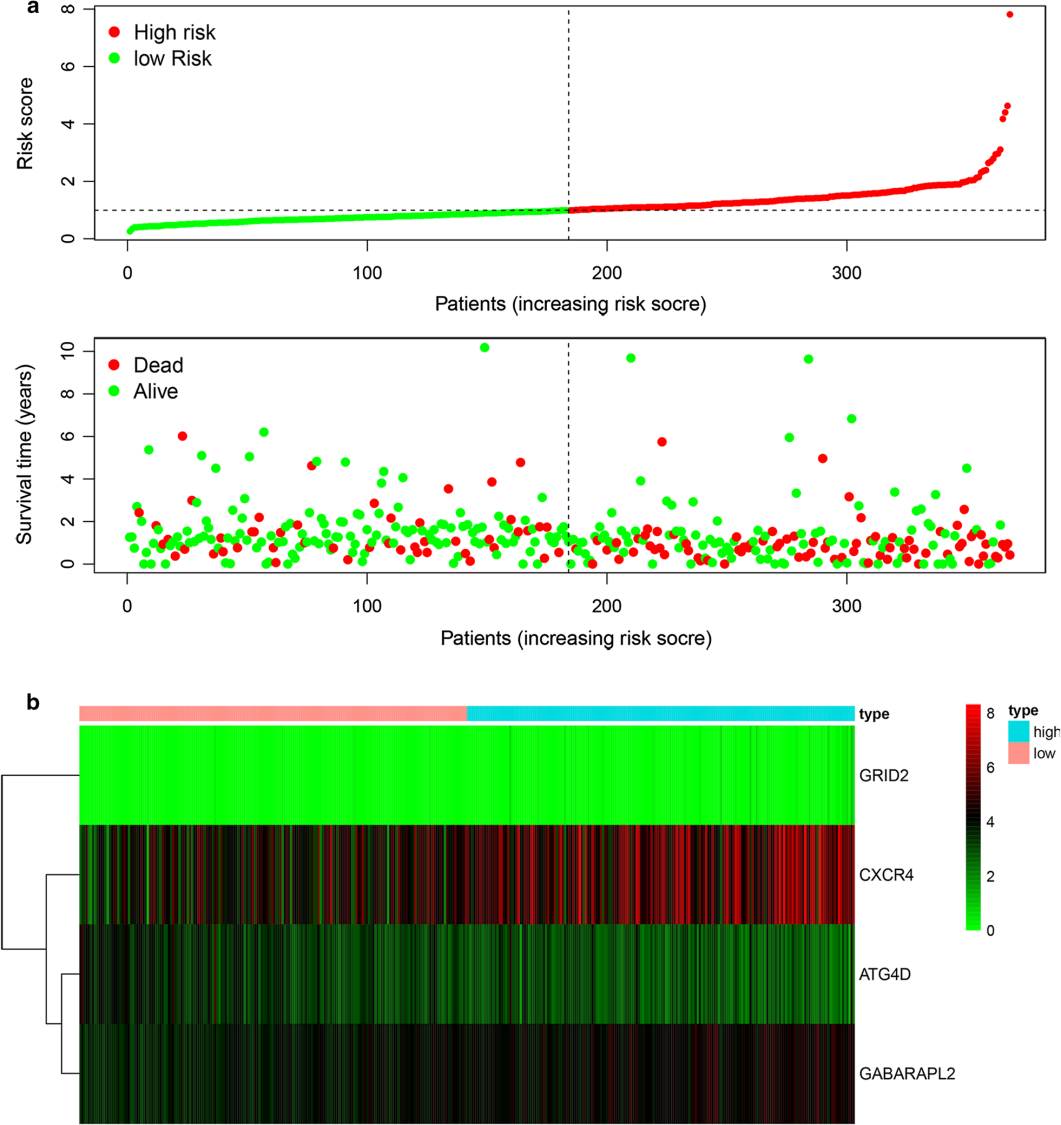
Characteristics of prognostic gene signatures. **a** Distribution of risk score and patient survival time, and status of STAD. The black dotted line is the optimal cut-off value for dividing patients into low-risk and high-risk groups. **b** Heat map of autophagy-related gene expression profiles in the prognostic signature of STAD

The risk score of each patient was calculated on the basis of the relevant mRNA expression level and risk coefficient of each ATG. The risk score is used to forecast the prognosis of gastric cancer, and the median risk score is the critical value to divide patients into high-risk and low-risk groups. Heatmap was drawn to show gene expression profiles in high-risk and low-risk STAD groups (Fig. 4b). The genes with HR > 1 (GRID2,GABARAPL2, CXCR4) are considered to be dangerous genes, while the gene with HR<1 (ATG4D) to be a protective gene (Fig. 4b). As shown in the Fig. 4b, patients in the high-risk group have more possibilities to express risk genes. In contrast, patients in the low-risk group have a disposition to express the protective gene (Fig. 4b). Figure 4a shows the distribution of risk scores in patients with gastric cancer and the relationship between risk scores and survival time.

### Autophagy as an independent prognostic factor

**Fig. 5:**
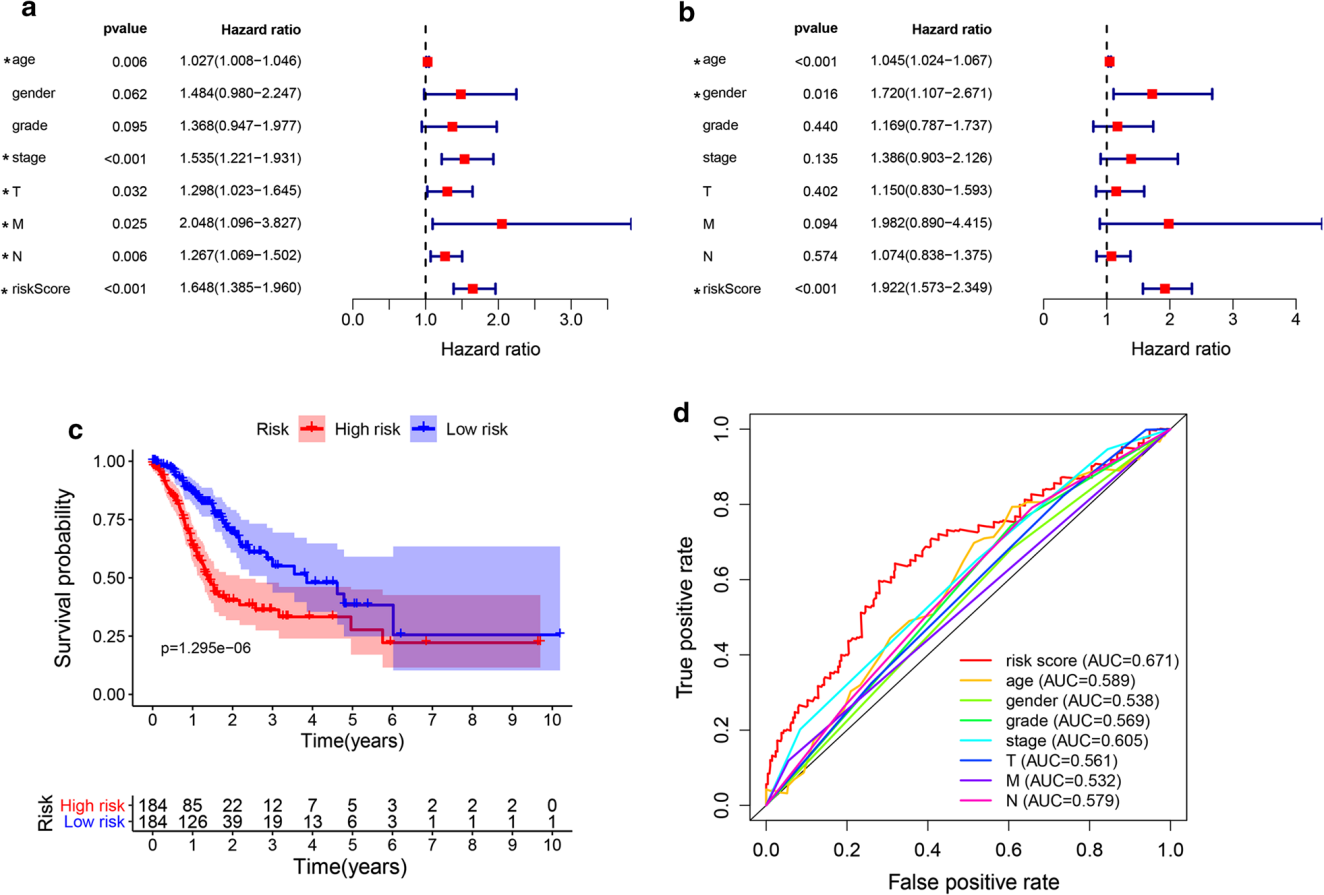
Autophagy-related gene signatures are significantly associated with gastric cancer survival. **a** Univariate Cox regression analysis. Forest plot of associations between risk factors and the survival of STAD. **b** Multiple Cox regression analysis. The autophagy-associated gene signature is an independent predictor of TCGA-STAD. **c** Kaplan–Meier analysis of TCGA gastric cancer patients was stratified by median risk. High risk scores are associated with general poor survival of TCGA-STAD. **d** Multi-index ROC curve of risk score and other indicators

We assessed the prognostic value of risk scores. For TCGA-STAD, the risk score in univariate analysis was significantly correlated with overall survival (OS) (HR = 1.648, 95% CI = 1.385–1.960, *P *< 0.001) (Fig. 5a). Multivariate analysis showed that the risk score was an independent prognostic indicator (HR = 1.922, 95% CI = 1.573–2.349, P < 0.001) (Fig. 5b). The Kaplan–Meier cumulative curve showed that the survival time of patients with low-risk score was significantly longer than that of patients with high-risk score (Fig. 5c). The AUC of risk score was significantly larger than that of other indicators, which proved that the Cox model had better ability to predict prognosis than other individual indicators.

### Construction and verification of nomogram

**Fig. 6:**
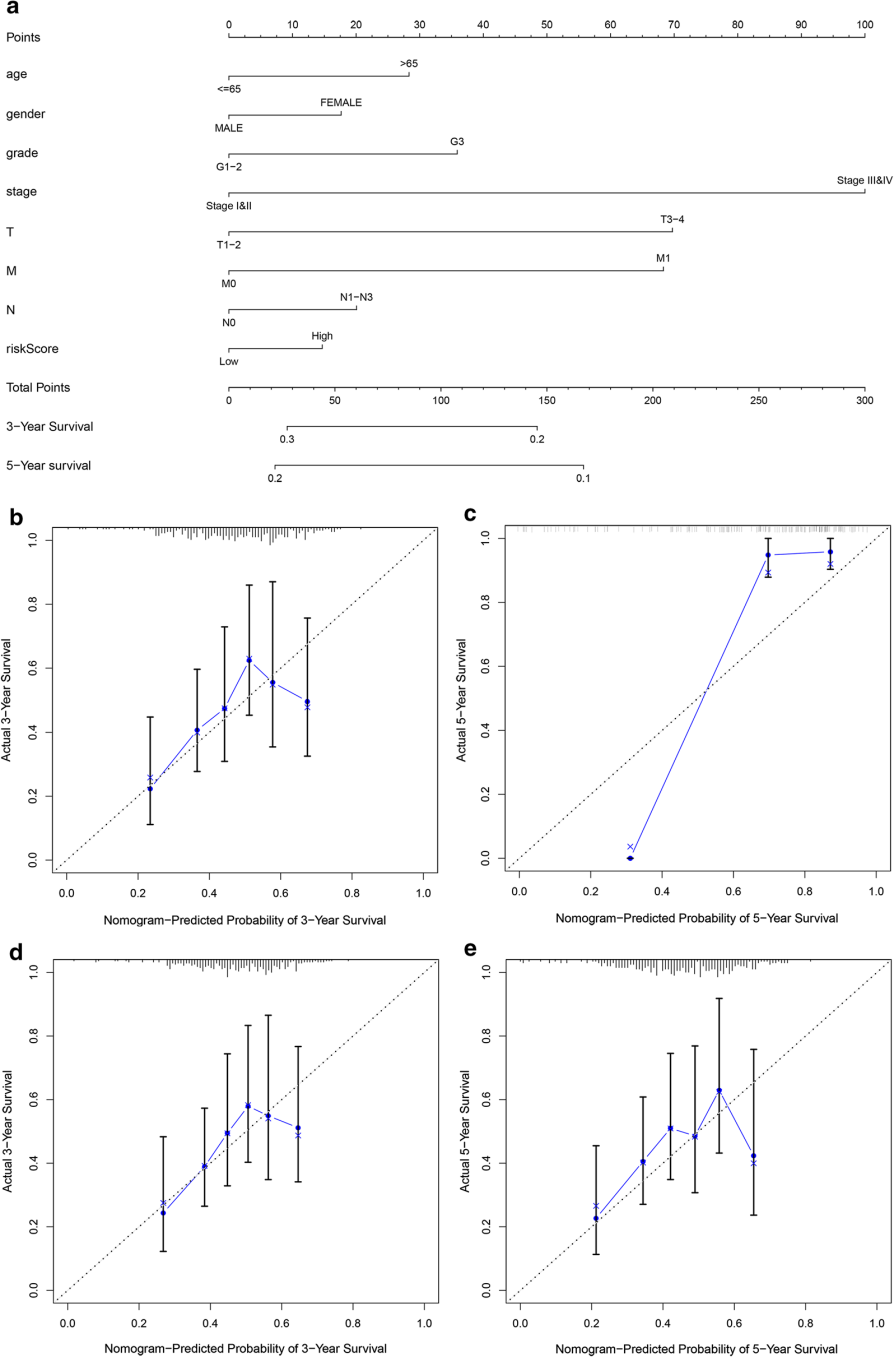
The nomogram can predict the prognosis probability in STAD. **a** A nomogram of the STAD cohort (training set) used to predict the OS. (B-C) Calibration maps used to predict the 3-year (**b**) and 5-year survival (**c**) in the training set. Calibration plots for 3-year (**d**) and 5-year survival (**e**) in the GSE62254 gastric cancer cohort (test group). The x-axis and y-axis represent the predicted and actual survival rates of the nomogram, respectively. The solid line represents the predicted nomogram, and the vertical line represents the 95% confidence interval

Nomogram is a powerful tool, which has been applied for quantifying individual risk in the clinical environment by integrating multiple risk factors. By combining four autophagy gene features, we performed nomogram to predict the possibility of 3-year and 5-year OS. As shown in Fig. 6a, the score assigned to each factor is proportional to its risk contribution to survival. The indication of calibration curve matches well (Fig. 6b, c). Nomogram has been validated in the GSE62254 gastric cancer dataset, and the 3-year and 5-year calibration curves are respectively shown in Fig. 6d and e.

## Discussion

Autophagy is a highly conserved evolutionary process in eukaryotic cells, which is involved in a series of cell homeostasis processes. There are three types of autophagy: Macroautophagy, microautophagy and chaperone mediated autophagy. Macroautophagy is the only autophagy that can degrade organelles, which we usually call autophagy. Autophagy related genes LC3, Beclin 1 and ATG5 are all biomarkers of autophagy, which are involved in autophagy regulation [16–18].

Many studies have shown that autophagy protein is closely related to the prognosis of GC patients. The high expression of ATG5 is closely related to the poor prognosis and drug resistance of gastric cancer [19]. It has been found that the disease-free survival rate and the overall survival rate of patients in the Beclin 1 high expression group are significantly increased [20]. However, some studies have come to the opposite conclusion [21]. Considering the importance of autophagy in gastric cancer, we can reasonably speculate that autophagy related genes have broad prospects in the prognosis evaluation of gastric cancer, and the multi gene signature generated by various algorithms will be better than a single molecule in the prediction of GC OS.

In this study, we analyzed the mRNA expression of 204 ATGs in the TCGA gastric cancer dataset. Single factor Cox regression analysis showed that 10 genes were related to the survival of STAD. We used LASSO regression to develop eight prognostic markers for the TCGA-STAD cohort. Finally, the signature of four genes was established by multivariate Cox regression. The risk score of each patient can be obtained by calculating the mRNA expression level and risk coefficient of the selected gene. In the TCGA-STAD cohort, risk scores significantly stratified patient outcomes. More importantly, in two independent geo gastric cancer datasets within the STAD, the prognostic power of the 4-gene signature was verified. Gene signature is often applied to forcast the prognosis of a variety of tumors in the past few years [22], which is even better than TNM staging and histopathological diagnosis in some extent [23]. Gene signatures based on ATGs have been reported in a variety of cancers, such as serous ovarian cancer, breast cancer, colon cancer and glioma [24–27]. For example, Liu et al. recently reported a 14 autophagy related signature based on relapse free survival in patients with non-small cell lung cancer [28].

In previous experiments, it has been shown that the genes contained in the signature are all related to cancer. The GluD2 protein encoded by GRID2 is a member of the ionic glutamate receptor family that mediates excitatory synaptic transmission [29]. Research by Ngollo et al. Showed that GRID2 interacts with H3K27me3 in prostate cancer and is significantly overexpressed. [30] The protein encoded by ATG4D belongs to the ATG4 mammalian family (four cysteine proteases, ATG4A4D class), which is closely related to autophagosome maturation and apoptosis pathways [31]. Gil et al. research shows that ATG4D can play a role as a tumor suppressor in the development of colorectal cancer [32]. Similarly, ATG4D’s defective expression will eliminate autophagy and promote the growth of human uterine fibroids [31]. The members of the GABARAP (gamma-aminobutyric acid hand-related protein) family (GABARAP, GABARAPL1/GEC1 and GABARAPL2/GATE-16) are one of the subfamilies of the ATG8 family of proteins, and are closely related to the intracellular transport of receptors and the autophagy pathway [33]. GABARAP is described as being down-regulated in cancer, and high expression is associated with a good prognosis [34]. The study of Y et al. Showed that GABARAP is overexpressed in colorectal cancer, which is related to the shortened survival time of patients, which shows the prognostic significance of GABARAP [35]. Moreover, the expression of ATG4 isoforms such as ATG4D can regulate post-translationally activated LC3/GABARAP family proteins [32], which further verifies the internal connection of genes contained in gene signatures. CXC type 4 chemokine receptor (CXCR4), also known as fusion protein (Fusin) or CD184, plays a role in cell proliferation and migration of cells [36]. Yu et al. Found that miR-125b induced by CXCL12/CXCR4 axis promoted invasion and conferred 5-fluorouracil resistance in colorectal cancer by enhancing autophagy [37]. The CXCR4/mTOR signaling pathway is also thought to play a role in promoting migration and inducing autophagic cell death in the peritoneal diffusion of gastric cancer cells [38].

Bioinformatics enrichment analysis showed that 28 differentially expressed autophagy related genes(DE-ATGs) were mainly related to cell growth, positive regulation of cell protein localization, neuron death, regulation of cell growth, platinum drug resistance, apoptosis and p53 signaling pathway in STAD. Interestingly, Huang’s study found that autophagy plays a vital role in the platinum drug resistance of tumor cells [39]. In tumor treatment, apoptosis tolerance is an important mechanism for tumor drug resistance. Autophagy can prevent apoptosis induced by antitumor drugs and promote tumor drug resistance. However, autophagy cell death may be a death mode of apoptosis tolerant tumor cells, Autophagy has double effects on drug resistance of tumor cells [40]. There is also a lot of evidence implying the interaction between autophagy and apoptosis [41]. Autophagy may promote or hinder apoptosis.

Autophagy inhibited apoptosis when the environmental conditions were less affected. However, when autophagy causes excessive consumption of intracellular proteins and organelles, resulting in the inability of cells to survive, the cells will turn into apoptosis. In some cases, autophagy can also cause cell death. It is worth mentioning that autophagy and apoptosis involve many apoptosis related proteins, such as p53 and BH3 only proteins [32]. In the early stage of cancer cell formation, autophagy can inhibit the formation of cancer cells; after cancer cell formation, cancer cells use autophagy to promote the survival of cancer cells and inhibit cell apoptosis, which may lead to the resistance of cancer cells to chemotherapy drugs. Therefore, if we inhibit autophagy during chemotherapy, it will be beneficial to enhance the therapeutic effect.

Finally, we developed a nomogram to predict individual clinical outcomes including these four autophagy-related gene signature, age, gender, grade, stage, TNM staging and risk score to construct a nomogram to predict the 3-year and 5-year survival of gastric cancer patients. Consistently, the calibration chart shows that the signature can more accurately assess the survival of gastric cancer patients. However, due to the lack of sufficient cases, we were unable to evaluate the predictive power of autophagy gene signatures in other independent gastric cancer data sets. In addition, other potential prognostic variables related to OS in GC, such as neutrophil-to-lymphocyte ratio (NLR), should also be studied. In addition, the expression and prognostic role of these four genes in gastric tissue need further study.

It should be admitted that our research inevitably has some limitations. First, our work is retrospective, not prospective; in addition, some other key clinical pathological features, such as the number of lymph nodes, are not included in the nomogram. Finally, the mechanism and interrelation of autophagy-related genes contained in gene signatures need further study.

## Conclusion

In summary, our study established a novel 4-gene signature and nomogram to forcast the prognosis of GC patients, which may contribute to the clinical decision-making of individual therapy.

## Supplementary information

**Additional file 1.** Clinicopathologic features of the patients in TCGA-STAD.

**Additional file 2.** 232 autophagy related genes obtained from Human Autophagy Database.

## Data Availability

The datasets used and/or analyzed in the present study are available from the corresponding author on reasonable request.

## Abbreviations

GC: Gastric cancer
ATG: Autophagy-related gene
DE-ATG: Differentially expressed autophagy-related genes
LASSO: The Least Absolute Shrinkage and Selection Operator (LASSO)
TCGA: The Atlas Cancer Genome
OS: Overall survival
DFS: Disease-free survival
GO: Gene Ontology
KEGG: Kyoto Encyclopedia of Genes and Genomes
PPI: Protein–protein interaction
GEO: Gene Expression Omnibus
BP: Biological process
CC: Cell components
MF: Molecular functions
STAD: Stomach adenocarcinoma
