# Supplementary material for: Identification and validation of an individualized autophagy-clinical prognostic index in gastric cancer patients
Source: Cancer Cell Int. 2020 May 20;20:178. doi: 10.1186/s12935-020-01267-y (PMC7240997; doi:10.1186/s12935-020-01267-y)
Supplement: Supplementary file 1 — Additional file 1. Clinicopathologic features of the patients in TCGA-STAD. [file 12935_2020_1267_MOESM1_ESM.doc]

**Additional file 1.** Clinicopathologic features of the patients in TCGA STAD

| Characteristics | N=375(%) |
| --- | --- |
| Gender |  |
| Male | 241(64.26) |
| Female | 134(35.73) |
| Age(year) |  |
| ≤60 | 121(32.27) |
| ＞60 | 254(67.73) |
| T stage |  |
| T1 | 19(5.07) |
| T2 | 80(21.33) |
| T3 | 168(44.80) |
| T4 | 100(26.67) |
| N stage |  |
| N0 | 111(29.60) |
| N1 | 97(25.87) |
| N2 | 75(20.00) |
| N3 | 74(19.73) |
| M stage |  |
| M0 | 330(88.00) |
| M1 | 25(12.00) |
| TNM stage |  |
| I | 53(14.13) |
| II | 111(29.60) |
| III | 150(40.00) |
| IV | 38(10.13) |
| Histological grade |  |
| G1 | 10(2.67) |
| G2 | 137(36.53) |
| G3 | 219(58.40) |
